# Comprehensive history of 3-year and accelerated US medical school programs: a century in review

**DOI:** 10.1080/10872981.2018.1530557

**Published:** 2018-10-30

**Authors:** Christine C. Schwartz, Aparna S. Ajjarapu, Chris D. Stamy, Debra A. Schwinn

**Affiliations:** Departments of Anesthesia (DAS), Biochemistry (DAS), Pharmacology (DAS), University of Iowa, Iowa City, IA, USA

**Keywords:** Review, 3-year medical school, accelerated, curriculum, US

## Abstract

Within the context of major medical education curricular reform ongoing in the United States, a subset of schools has re-initiated accelerated (3-year) medical education. It would be helpful for education leaders to pause and consider historical reasons such accelerated medical schools were started, and then abandoned, over the last century to proactively address important issues. As no comprehensive historical review of 3-year medical education exists, we examined all articles published on this topic since 1900. In general, US medical educational curricula began standardizing into 4-year programs in the early 1900s through contributions from William Osler, Abraham Flexner, and establishment of the American Medical Association (AMA) Council of Medical Education (CME). During WWII (1939–1945), accelerated 3-year medical school programs were initiated as a novel approach to address physician shortages; government incentives were used to boost the number of 3-year medical schools along with changed laws aiding licensure for graduates. However, this quick solution generated questions regarding physician competency, resulting in rallying cries for oversight of 3-year programs. Expansion of 3-year MD programs slowed from 1950s to 1960s until federal legislation was passed between the 1960s and the 1970s to support training healthcare workers. With renewed government financial incentives and stated desire to increase physician numbers and reduce student debt, a second rapid expansion of 3-year medical programs occurred in the 1970s. Later that decade, a second decline occurred in these programs, reportedly due to discontinuation of government funding, declining physician shortage, and dissatisfaction expressed by students and faculty. The current wave of 3-year MD programs, beginning in 2010, represents a ‘third wave’ for these programs. In this article, we identify common societal and pedagogical themes from historical experiences with accelerated medical education. These findings should provide today’s medical education leaders a historical context from which to design and optimize accelerated medical education curricula.

## Introduction and purpose

Over the last decade, US medical school education leaders have embarked on innovative curriculum reform. In addition to integrating clinical and biomedical science teaching over a 4-year continuum and adding active learning, some medical schools have begun experimenting with delivering medical education in a more comprehensive period, specifically 3 years. This movement has created some controversy, particularly since it has been tried and abandoned twice before in US history in various contexts. Yet in researching background information on accelerated medical education in the United States, there appears to be no comprehensive historical review of 3-year MD schools, since articles generally focus on specific schools and/or specific time periods. Given that we can learn from the past, our research group embarked on an exhaustive historical review of manuscripts published about accelerated allopathic MD education programs in the United States over the last century. Our purpose is to examine reasons medical schools had for choosing, and then abandoning, accelerated 3-year medical education over time. Reasons for embarking on such programs are not always educational (e.g., an acute need for physicians during war, or desire to decrease overall student debt), so we undertook this review with the broadest possible lens to highlight social, political, structural, and educational milieu for decisions made. This comprehensive review summarizes conditions that favored establishment of 3-year MD programs over time, as well as issues and concerns encountered. It therefore should provide a helpful context for education leaders as they reflect on whether 3-year accelerated MD education fits their unique institutional environment, and if so, how to optimize success.

## Detailed history

### Initial medical curriculum standardization: 1800s–1930s

During the 1800s the majority of medical schools in the United States were small, for-profit, non-university affiliated, and had wide-ranging non-standardized curricula and educational goals. In 1847, the American Medical Association (AMA) was established and later the AMA Council of Medical Education (CME) was initiated, ultimately providing a standardized medical school curriculum in 1904 [1]. A founding father of this new curriculum was the Canadian physician, Sir William Osler, who introduced rigorous clinical clerkships for medical students in the 1890s. In the early 1900s, Abraham Flexner was commissioned by the Carnegie Foundation to review medical education in the United States [2]. Impressed by the education at John Hopkins, headed by Osler, Flexner emphasized that 2 years of science education should precede Osler’s intensive clinical training, comprising a total of 4 years of medical school . The Flexner report of 1910 (pdf of the original document is available online through the Carnegie Foundation archives) was revolutionary in providing a critical framework for medical education, essentially resulting in a model that became standardized in a wave of consolidated university affiliated medical schools. ‘By 1934 all but eight of the 38 approved graduate schools of medicine were related to university medical schools’ [3]. Post WWI (1918), rapid expansion of medical techniques and capabilities occurred, leading to physician-teacher supervised patient care immediately following medical school (internship) [2]. By 1930, premedical science requirements had also become clearly established, including two semesters each of general chemistry, physics, and biology, and one semester of organic chemistry.

### Introduction of the 3-year accelerated medical curriculum: 1930s–1940s

During World War II (1939–1945), the nation faced physician shortages at home and abroad, so 3-year accelerated medical school programs were introduced as a proposed solution to educate physicians faster [4]. The Medical College of Virginia was one of the first schools to have a 3-year accelerated MD program during World War II [5]. Pressured with solving physician shortages, the US federal government urged universities and medical colleges to adopt 3-year accelerated medical programs more broadly. Indeed, the Federation of State Medical Boards revised laws and regulations governing licensure so students graduating in 3 years could legally practice medicine. Because very little (if any) time was given to appropriately develop these new 3-year curricula, the AMA Board of Trustees organized a liaison committee to aid in overcoming difficulties encountered. In addition, the AMA’s Council on Medical Education and Hospitals took responsibility to oversee inspections to maintain high standards of medical education due to concerns of deterioration of quality with accelerated medical programs [6]. Interestingly, once military physicians were discharged into civilian status, many recognized their limited knowledge so ‘refresher’ courses gained attraction. In a survey sent to a large sample of physicians in the military, nearly 60% (12,534/21,029) wanted to take long refresher courses (6-months or more) and this led to pressure to develop more post graduate medical education (GME) programs. ‘By the academic year 1955–1956, 63 of the 85 approved or developing medical schools were engaged to some degree in post-graduate medical education programs’ [3]. By the end of the 1940s, GME materialized more formally and hospital based residencies became the path to specialization [7]. The influence of the National Institutes of Health (NIH), with its government-funded research grants, aided early development of physician scientists.

### Federal legislation changes and introduction of alternative accelerated pathways: 1950–1971

Between 1950 and 1960, there was little expansion or development of new 3-year programs compared to the previous decade. Mounting concern regarding physician shortages plagued the 1960s and, in response to these concerns, federal legislation was passed to support expansion of health profession schools. In 1963, the Health Professions Education Assistance Program was implemented to provide support for the health professions. This support included construction aid for health profession schools (allopathic and osteopathic medicine, dentistry, veterinary, optometry, pharmacy, podiatry, public health, and nursing) as well as providing loans to students of dentistry, allopathic MD and osteopathic DO medicine [8].

New amendments to this legislation were made in 1965, which included basic and special improvement grants, new requirements for construction funding (e.g., requiring buildings be used for education for a longer period of time than previously required), increasing funding for student scholarship programs and increased maximum loan amount ($2,500 maximum scholarship per student/year and $2,500 maximum annual loan per student), and creating loan forgiveness programs. Basic grants aimed to expand enrollment in health professions schools by providing a lump sum to each approved school in addition to extra money per student [9]. Special grants stimulated accreditation and established special programs [8,9]. The Health Professions Education Assistance Program increased authorization of funding to programs such as the National Health Service Corps, which required schools to ensure that an increasing percentage of first-year residencies in affiliated hospitals were reserved for primary care training in order to receive capitation support. The 1976 version of the Health Professions Education Assistance Act authorized funding to increase the capacity of existing Area Health Education Centers, which provided clinical training programs for health professionals in underserved areas [10]. These grants successfully increased student enrollment and graduation [8].

Due to the concern about healthcare worker shortages, a flurry of other additional federal legislations were passed between 1965 and 1971, including the Allied Health Professions Personnel Training Act in 1966, Health Training Improvement Act in 1970, Nurse Training Act in 1971, and Comprehensive Health Manpower Training Act in 1971 [8]. The Comprehensive Health Manpower Training Act was notable for elevating federal commitment to training healthcare workers, giving new urgency to training family practitioners, increasing numbers of minorities in health professions, and alleviating shortages in underserved areas. This piece of legislation was crucial to the expansion of 3-year medical programs in the 1970s [8].

Of note, during the 1950s and 1960s, other innovative alternative pathways to an MD degree were introduced. These programs were designed to shorten the overall time needed to train a physician and included 3 + 3 programs that combined 3 years of undergraduate courses with 3 years of medical school (BA-MD programs). In the 1960s, 60 students were admitted into BA-MD programs nationally. That number grew to around 400 students per year by 1976 [11]. Due to improved high school and undergraduate collegiate programs developed during the 1950s and 1960s, medical students were able to start at a more advanced level than had been possible in the past, which enabled 3-year medical school programs to be more achievable than they had been during the WWII era. Indeed, by 1967 over 40% of medical school applicants had some exposure to biochemistry [12]. Students in BA-MD programs generally succeeded academically. For example, 50% of students who entered Boston University’s BA-MD program received their BA degree with honors and ≥10% received their MD degree with honors [13]. A review of performance outcome data of students in combined BA-MD programs from 1966 to 1996 showed no difference in competency from traditional medical school students as determined by scores on standardized medical board exams and clinical performance. These results demonstrated that students could be selected from high school to become successful physicians [14]. The 1960s also saw a trend toward larger medical school class sizes. For example, between 1960 and 1970, the University of Minnesota Medical School increased its freshman class from 164 to 227 students [15].

### A spike in the number of 3-year accelerated medical school programs: 1970s–1980s

In addition to addressing physician shortages during the Vietnam War era (1955–1975), much like during WWII, the 1970s had an additional factor that led to the rapid development of 3-year programs: the desire to reduce student debt. A successful push to reduce student debt may be revealed in decreased numbers of medical students working for pay in the 1970s. ‘In 1963, 45% of medical school seniors worked an average of 16 hours a week; in 1971, fewer (34%) of seniors worked an average of 14 hours a week; and, by 1974, only 26% of seniors worked an average of 10 hours per week’ [16]. National discussion regarding reducing student debt, the need to address physician shortages, and government financial incentives were reported as major contributors to increased enrollment in 3-year programs from 1970 to 1973 [17]. Indeed, during this time, enrollment in 3-year accelerated MD programs increased 387%, from 671 students in the 1970–1971 academic year to 2,597 in 1973–1974 [8] (out of a total of 15,000 medical graduates per year [18]). The greatest increase in 3-year MD enrollment was seen after implementation of the 1971 Comprehensive Health Manpower Training Act, with first year medical school enrollment increasing from 1,080 in 1972–1973 to 2,273 during the subsequent academic year [8]. Under the Comprehensive Health Manpower Training Act of 1971, federal capitation funding provided a bonus of $2000 per student graduating in 3 years [19]. Given this context, by 1973 approximately one-third (*n* = 33) of all US medical schools offered a 3-year MD path to graduation [9,20].

Positive attitudes and interest in developing accelerated medical programs during the 1970s resulted in increased overall medical school enrollment, including 3-year programs, and expansion in a number of schools offering other accelerated pathways. There were advantages for students that facilitated expansion of accelerated programs as well. In this regard, it should be noted that rising competition for admission into medical school occurred from 1970 to 1975; in 1970, there were 25,000 applications for 11,350 openings. By 1975, there were 43,000 applications for 15,000 places, which caused the percent of matriculates to fall from 45% to 35% (1970 to 1975, respectively) [11]. Students may have reasoned that early admission to accelerated MD programs might be a way of avoiding increasing competition within the broader general admission pool [11]. This was also beneficial for medical schools since outstanding candidates were attracted into new 3-year MD programs, and these students in turn boosted admission metrics and performance of their peers, an important component of overall institutional prestige [11]. Faculty attitudes toward 3-year MD programs varied, interestingly, by specialty. When Ohio State University School of Medicine transitioned from a 4-year program to a 3-year MD program in 1970, a study of faculty attitudes determined that family medicine and preventive medicine faculty were most positive toward 3-year MD programs [20].

### Discontinuation of 3-year accelerated MD programs: late 1970s–1980s

Many of the 3-year accelerated medical school programs introduced and developed in the 1970s did not stay open long-term. Academic stress was high and about 25% of students in accelerated MD programs voluntarily extended their education by 1 or 2 years [9]. Additionally, students and faculty cited feeling pressured by compression of the material; students completing programs stated that they felt ‘exhausted,’ and faculty felt dissatisfied with trying to teach ever-expanding medical knowledge into a shortened timeframe [9]. Other issues cited with 3-year curricula included under-representation of family medicine, rural medicine, and gerontology rotations, as well as lack of in-depth ethics and substance abuse training. In addition, there was limited exposure to faculty members who would ultimately prepare residency recommendation letters, little time for students to interview with residency programs, and minimal vacation time [17]. Perhaps most importantly, accelerated MD programs did not increase net physician output, which was one of the main goals of starting such programs [9]. Without an increase in class size, 3-year medical programs created a bolus of physicians when the first class of 3-year students graduated along with fourth year students. Thereafter, the net number of students graduating remained stable (reached steady state), without further increase.

It is important to note that pedagogy for a 3-year MD curriculum is distinct and therefore should go beyond compression of a school’s 4-year curriculum. This was sometimes learned the hard way in the 1970s. As an example, the University of Arizona went from a 4-year to a 3-year MD program in 1972, and then converted back to a 4-year program in 1977. Arizona had compressed 72 weeks of basic science (pre-clinical) material into 56 weeks initially, and then increased it to 64 weeks in the second class and subsequent classes in their 3-year MD curricula. Medical students at Arizona had the option to extend their schooling to four or more years if needed. One-third of students in the first three classes at Arizona extended their time beyond 3 years, and students who took this option used the majority of additional time to slow down the pace of basic science learning rather than choosing more time to explore medical specialties [17]. Unfortunately, students who extended their time beyond the originally committed 3 years were often stigmatized and perceived by faculty as ‘weak or deficient’ [9,18].

Other schools felt pressure from their compressed MD curriculum as well. At Ohio State, the mean satisfaction score of faculty participating in the 3-year curriculum was 60.5 out of 100 (range of 34–93), with 50% of faculty favored returning to a 4-year MD program [20]. Although studies showed no major difference in performance between 3-year and 4-year students, as measured by USMLE scores and residency match results, by the end of the 1970s most 3-year MD programs were discontinued in favor of 4-year curricula [13,17,21,22]. Indeed, after the 1973–1974 academic year, a persistent decline in enrollment in these programs occurred, with 2,434 3-year MD students in 1974–1975 declining to 1,455 in 1978–1979 [23]. Overall reasons given for ending 3-year MD programs included discontinuation of federal funding that eliminated financial incentives, declining physician shortages, and overall dissatisfaction with the accelerated time frame [22].

### Addressing the length of physician training with growth of 3 + 3 MD-residency programs: 1980s–2000s

Although there was dissolution of most 3-year MD programs by the end of the 1970s, over the next few decades, growth occurred in combined MD-residency programs in an attempt to reduce the overall length of training of a physician. From 1975 to 2000, the number of residents in the United States had more than doubled, from 37,140 to 98,806 [18]. Some medical schools with 4-year curricula began allowing students to waive the fourth year if they completed a rotating internship [17]. In the 1980s and 1990s, 25 US medical schools had family medicine 3 + 3 (MD-residency) programs in addition to several in internal medicine 3 + 3 [21]. Interestingly, family medicine 3 + 3 graduates were more likely to be chosen as chief residents than traditional (4 + 3) graduates. Most of these 3 + 3 programs ended, however, primarily due to GME accreditation issues [22].

### Current state of 3-year accelerated medical school programs: 2010–2017

With the turn of the century, vast reforms have occurred in medical school curricula. Since 2005, 75% of US medical schools have initiated innovative pedagogy, including active learning and enhanced integration of clinical and biomedical sciences [2]. This shows general recognition that revised curricula that optimize adult learning and incorporate updated medical knowledge are needed for physicians to succeed in the twenty-first century. As part of curricular reform, since 2010, 3-year MD programs have re-emerged in nine allopathic medical schools, with 80% of these schools focusing on primary care for their 3-year students [21]. Given the limited time available in 3-year MD curricula to travel for residency interviews, almost all of these programs offer their students guaranteed local residency positions [21,24,25].

### Summary of historical trends

**Figure 1.**
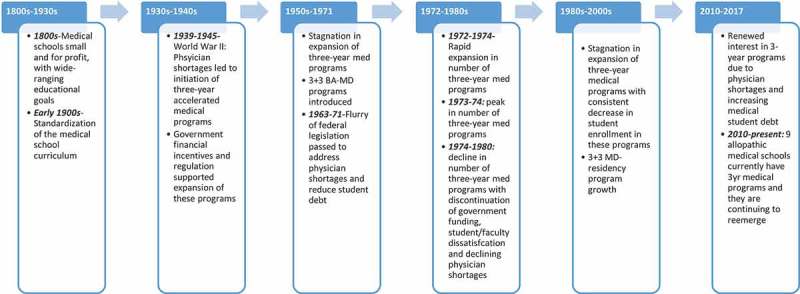
History of events impacting creation and dissolution of 3-year MD programs in the United States over the last century.

**Figure 2.**
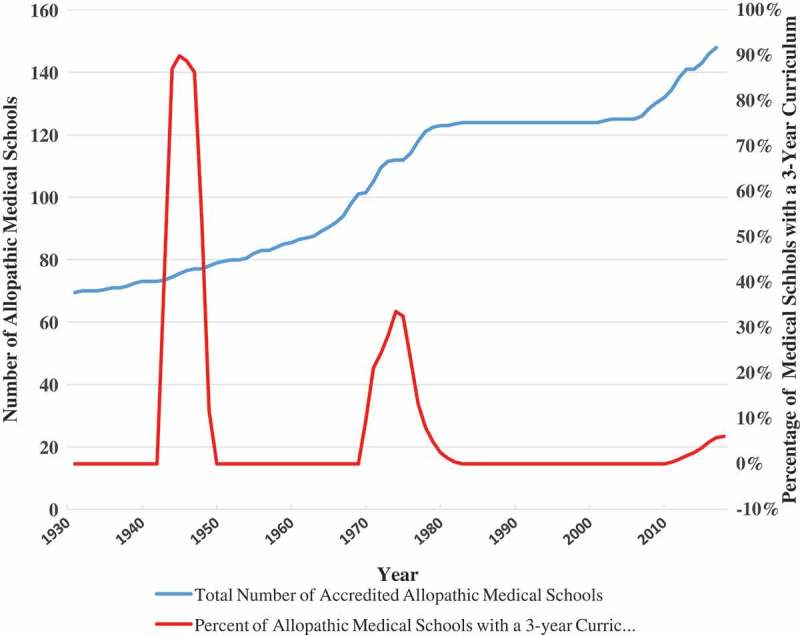
Estimated percentages of allopathic medical school 3-year curriculum overtime.

Figure 1 summarizes historical events impacting creation and dissolution of 3-year MD programs in the United States over the last century. Figure 2 documents the rise in total medical students from 1930 to 2017 juxtaposed against percentages of accredited allopathic medical schools with 3-year curriculum over the same period [9,12,20,22–38]. As can be seen in Figure 2, the greatest percentage of medical schools with a 3-year program occurred in the WWII era (≈90%) followed by the 1970s (≈40%). The most recent resurgence of medical schools with 3-year curricula is far less pronounced compared to spikes in the 1940s and 1970s.

## Emerging themes across time

Two major themes (learning lessons) emerge across the last century when examining accelerated 3-year medical education. The first theme is that financial benefit of 3-year medical education (e.g., decreased student debt and lower medical education costs) is an ideal that is not as straightforward in reality as originally conceived and depends on broader context of institutions. A second (pedagogical) theme is the importance of developing new, integrated, and streamlined curricula for 3-year medical education, distinct from the approach to 4-year medical education. Each of these findings is discussed below.

### Financial advantages and costs of accelerated/3-year medical schools

Medical student debt (combined undergraduate and medical school) remains an important problem in the United States. From 1990 to 2003, medical school tuition and fees grew between 83% and 167% [35]. It should be noted, however, that it is erroneous to assume that 3-year MD programs decrease medical school debt by simply eliminating 1 year of tuition since the situation is more complex. In fact, some 3-year medical schools charge the same tuition as 4-year schools [28,36]. This is because currently the US LCME requires 130 weeks for a MD degree, which is achieved in 3-year programs by adding classes during summers and by decreasing both vacation time and residency interview time [22,37]. From an institutional perspective, such restructuring often requires a new set of teachers and other expenses. Some benefits do accrue, however, since many current 3-year programs today guarantee residency positions; this can lead to immediate $1000–$5000 savings to students in eliminating senior year interview expense [39].

Graduates of 3-year MD programs *do* acquire financial benefit by entering the workforce 1 year early compared to traditional medical students. This extra year allows an additional year of practice and resultant clinical income that could facilitate repayment of student loans. Financial benefit of 1 year gained in the workforce today has been reported to be $160,000 for general internists and $230,000 for internal medicine subspecialists [35]. This reality points out that the most effective financial means for decreasing the large financial burden of medical education may not lie in reducing the price of medical education *per se*, but rather by allowing earlier entry into the workforce so loans can be paid back sooner. This would be true whether acceleration occurs at the undergraduate, medical school, or residency level.

### 3-year medical school as a distinct pedagogy

Another theme present over time is that strong faculty and student dissatisfaction occurs with compression of a standard 4-year curriculum into 3 years since it is difficult to teach/learn such a large body of detailed material. This suggests that creating new, streamlined, and integrated curricula will be important for achieving success in accelerated medical education programs. Given current medical education curriculum reform efforts in the United States, and new understanding of effective approaches to adult learning, for the first time *proactive* intervention is possible in this regard. This is an important pedagogy lesson, and reality check (due to investments required), for leaders considering moving toward 3-year MD programs.

### One view of the future

Moving forward, a major (unrealized) advantage of accelerated 3-year MD programs may be in providing flexibility within current 4-year curricula by offering an opportunity to individualize training for a given student. With an explosion of technology undergirding all forms of education, synchronous and non-synchronous learning is now possible and this may result in enhanced satisfaction and flexibility for both students and faculty in these programs. Indeed, completing basic science and clinical training over 3 years may enable students to take advantage of other rich opportunities available in academic medical centers such as research, dual degrees, leadership training, and longitudinal team-based experiences otherwise not possible within older, more traditional (2 + 2) 4-year curricula. For students who prefer to focus solely on clinical medicine, ability to enter the work force 1 year early is a potential advantage since it helps with loan repayment and therefore can have important financial benefit.

Disadvantages in individualizing medical education (e.g., having two parallel 3-year and 4-year MD curricula in tandem within a given medical school), revolves predominantly around added cost. This is due to increased faculty resources needed without net added tuition, particularly in an era where modern innovations in medical school curricula already require significantly more faculty involvement for small group learning. In the absence of government subsidies, as occurred in the past when this pathway was implemented, such a view of the future may be difficult to sustain in today’s increasingly fiscally limited environment. However, from a pedagogical perspective, it is enticing to envision truly individualized education.

## Conclusion

Throughout the history of medical education in the United States, there have been three waves of interest and implementation of 3-year MD programs (WWII era, 1970s, and current). Common factors driving every period of growth include concern over addressing physician shortages. Historically, a reoccurring finding is that to truly address physician shortages, increasing class size of 3-year or 4-year medical school is needed. Other influences spurring growth of accelerated MD curricula have been governmental funding and, since the 1970s, an interest in reducing the cost of medical education. As stated above, financial incentive from accelerated medical programs lies predominantly in the ability of students to enter the workforce 1 year early, which can be effectively achieved by shortening several different educational time points. Reasons given for discontinuation of 3-year MD programs were elimination of external (government/state) funding, which waned in periods of declining concern over physician shortages, and dissatisfaction expressed by medical students and faculty participating in accelerated programs. In spite of these concerns, 3-year MD education graduates perform comparably with 4-year graduates on national examination scores.

Given expansion of technology used in teaching, and gradual transfer of medical school level classes to university undergraduate coursework, training physicians in a shortened duration may be more feasible nowadays compared to the past. For students who would like to complete alternative coursework such as research, 3-year MD programs can also provide a framework for individualization not accessible in traditional 4-year curricula. However, history also suggests caution. Standardizing 3-year curriculum is difficult and focused time and effort must be allotted to organizing such pedagogy if a long-term sustainable model is sought. By reviewing the context and issues encountered with 3-year MD programs in the past, it is the author’s hope that new programs can be designed in the most thoughtful and successful manner possible going forward.
